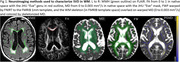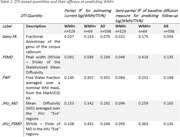# A Comparison of DTI‐Based Measures as Predictors of Future Cerebrovascular Degeneration

**DOI:** 10.1002/alz.093860

**Published:** 2025-01-09

**Authors:** Robert I. Reid, Scott A. Przybelski, Timothy G. Lesnick, Angela J. Fought, Sheelakumari Raghavan, Michael G. Kamykowski, Jonathan Graff‐Radford, David S. Knopman, Ronald C. Petersen, Clifford R. Jack, Prashanthi Vemuri

**Affiliations:** ^1^ Mayo Clinic, Rochester, MN USA; ^2^ Department of Quantitative Health Sciences, Mayo Clinic, Rochester, MN USA

## Abstract

**Background:**

There has been a recent proliferation of various quantities based on single shell Diffusion Tensor Imaging (DTI) for capturing overall cerebrovascular health, especially Small Vessel Disease (SVD) as a single number. The existing literature has gaps in the comparison of these measures, so we evaluated them as predictors of both current and future White Matter Hyperintensity (WMH) as a proxy of SVD.

**Method:**

Using baseline 3T MRI examinations (T1, DTI, FLAIR scans) of 598 participants (>60 years, amyloid negative, to limit to those on the SVD pathway) from the Mayo Clinic Study of Aging, we calculated five summary DTI quantities with varying degree of complexity. We estimated their partial R2 from cross‐sectional linear regression models and computed semi‐partial R2 from mixed models predicting WMH volume as a percentage of Total Intracranial Volume (TIV). All models were adjusted for age, sex, and scanner model, and the WMH and based were log‐transformed before fitting. We also conducted subset analyses with high (WMH+) vs. low (WMH‐) baseline WMH load (cut at 1.62%) to compare the performance of the measures in early vs. widespread WMH.

**Result:**

All MD‐based quantities computed on the whole brain, including FWF, performed similarly to each other, but Genu FA underperformed at explaining WMH variance (Table 1). PSMD (skeletonized or atlas based) was more sensitive in WMH+ but dramatically less so in WMH‐. In WMH‐ and overall cohort, FWF and JHU MD (which is a simple averaging of MD in the JHU atlas) performed well.

**Conclusion:**

All MD‐based measures performed similarly to each other in the whole dataset but dichotomizing by WMH load showed interesting differences. PSMD measurements are driven by the extreme end (95th percentile) of the MD distribution and therefore more sensitive in WMH+. Genu FA, a regional measure, was not useful for predicting whole brain WMH change. We noticed some variation in how the quantities are affected by scanner model (GE Signa HDxt and Discovery 750) which needs broader investigation. Simply averaging MD over WM avoids additional processing and is more sensitive to WM degeneration that is not (yet) severe.